# Characteristics Associated with Engagement in the Early Detection Cascade of Care for Psychosis at a College Counseling Center

**DOI:** 10.1007/s10597-024-01430-z

**Published:** 2025-01-05

**Authors:** Sam Barans, Justine L. Saavedra, David T. Lardier, Mauricio Tohen, Rhoshel Lenroot, Juan Bustillo, Dawn Halperin, Bess Friedman, Rachel Loewy, Cristina Murray-Krezan, Stephanie McIver, Annette S. Crisanti

**Affiliations:** 1 Department of Psychology, Drexel University, Philadelphia, PA USA; 2 Health Sciences Center, Department of Psychiatry and Behavioral Sciences, University of New Mexico, Albuquerque, NM USA; 3 Department of Psychiatry, University of California San Francisco, San Francisco, CA USA; 4 School of Medicine, Department of Medicine, University of Pittsburgh, Pittsburgh, PA USA

**Keywords:** Psychosis, Cascade, College, Early, Detection, Engagement

## Abstract

Using the Cascade of Care framework, we explored the demographic and clinical characteristics of students at six stages in an early psychosis detection program at a college counseling center, with a focus on the transition between stages with the highest disengagement. We detailed and compared the demographic and clinical characteristics of those who (1) completed the Prodromal Questionnaire-Brief (PQ-B, N = 1588); (2) met the PQ-B cutoff score (n = 486); (3) were referred for secondary phone screening (n = 404); (4) completed secondary phone screening (n = 198); (5) completed a Coordinated Specialty Care (CSC) eligibility assessment (n = 51); and (6) were enrolled in CSC (n = 21). Education level and gender identity were associated with engagement at multiple stages of the early detection cascade. Graduate education level, transgender or gender diverse gender identity, alcohol use, and depressive symptoms predicted student follow-through with referral to secondary phone screenings.

## Introduction

Since the passage of the Mental Health Block Grant in 2014, the number of Coordinated Specialty Care (CSC) programs for early intervention in psychosis has proliferated on account of their demonstrated efficacy (George et al., 2022). CSC programs across the United States aim to improve identification of individuals at risk of or actively experiencing a first episode of psychosis (FEP) and provide timely evidence-based services (George et al., 2022). As onset of psychotic disorders often occurs in late adolescence or early adulthood, college student populations are a key target for the early identification and treatment of psychosis (Hardy et al., 2018). Despite the number of CSC programs embedded within university healthcare systems or collaborating with college counseling centers (Read & Kohrt, 2022; EPINET, 2022), there have been few attempts to use the existing infrastructure for student-targeted early detection to examine areas for improvement in the identification and referral of college students to psychosis services.

Longer duration of untreated psychosis (DUP) has been associated with worse functional and clinical outcomes following treatment as well as heightened baseline symptomatology (Drake et al., 2020). The Cascade of Care framework, as modified for depression, exists to improve screening for psychiatric conditions by identifying implementation gaps at multiple intercepts in an individual’s pathway to care (Cox et al., 2016; Pence et al., 2012). To understand how to reduce DUP, Saavedra et al. (2023) used the cascade of care framework to identify six stages in the referral process for students at a university-based CSC program. The number of students at each stage were documented, consisting of the number of students who (1) completed the Prodromal Questionnaire-Brief (PQ-B; Loewy et al., 2011); (2) met the PQ-B Distress cutoff; (3) were referred for phone screening; (4) completed phone screening; (5) completed a CSC eligibility assessment; and (6) were enrolled in CSC. Disengagement throughout the identified stages may be influenced by program-level factors (e.g. pre-determined cutoff scores, clinicians’ decisions to refer, CSC program eligibility criteria) or student-level factors (e.g. completion of phone screen or CSC eligibility assessment). While attrition was observed between all stages, the highest rate of disengagement in the early detection cascade was observed between referral for and completion of phone screening (stages 3 to 4). Of the 404 students Saavedra et al. (2023) found referred for phone screening (stage 3), 49% (n = 198) of students followed through (stage 4). This suggests that student-level factors were associated with the highest disengagement rate observed in the early detection cascade.

The present study expands on the work of Saavedra et al. (2023) by describing the demographic and clinical characteristics of students throughout the cascade with the aim of identifying characteristics associated with disengagement between stages. As the highest rate of disengagement was observed between referral for and completion of a phone screening (a student-level factor), we chose a priori to focus on changes in student characteristics across the transition between stages 3 and 4. Understanding demographic and clinical characteristics associated with disengagement throughout the cascade of care may assist in targeting improvements for psychosis identification and services on college campuses.

## Methods

**Fig. 1:**
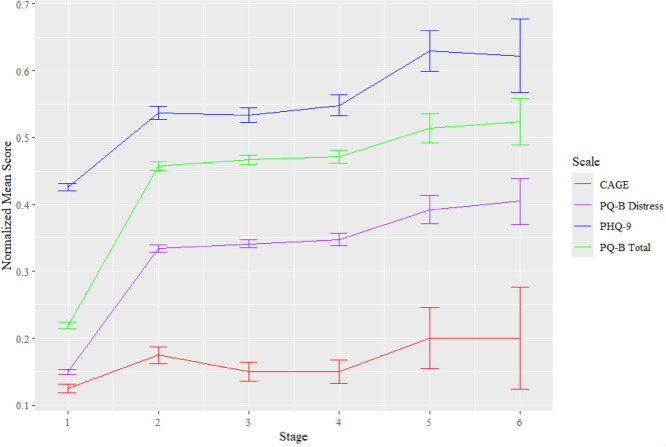
Clinical characteristics across the early detection cascade. *Note* Mean scores of clinical characteristics were calculated by averaging scores of all students who reached each given stage of the early detection cascade, then normalized (each respective measure was divided by the total number of points possible for that measure) so that all range from 0 to 1 for easier visual comparison. Error bars reflect one normalized standard error below and above the mean

This study was a secondary analysis of data summarized by Saavedra et al. (2023). Data were originally collected between August 1, 2020, and May 13, 2022. IRB approval was obtained for this study from the UNM Human Research and Protections Office (ID #19-118). All demographic and clinical characteristics described below were collected at student intake into the University of New Mexico (UNM) Student Health and Counseling (SHAC) center. At the time of the study, the UNM Department of Psychiatry and Behavioral Sciences’ CSC Program included clinics for clinical high-risk (CHR) and FEP. The CHR clinic served youth and young adults 12–25 years of age who met the criteria based on the Structured Interview for Psychosis Risk Syndromes (SIPS; McGlashan et al., 2001). The FEP clinic provided services to youth aged 15–30 years who were first diagnosed with affective or non-affective psychosis within 12 months prior to referral (Fig. 1).

### Study Population

The study population included 1588 students who presented to SHAC through self-referral and completed a PQ-B as a required part of the intake evaluation. SHAC provides basic counseling, evaluation by a psychiatrist, and/or screening and assessment for referral to specialized services as needed. The CSC Program at UNM is one such specialized service that students in need can be referred to.

At the beginning of our study, UNM had an undergraduate enrollment of 15,914 students and a graduate enrollment of 6067 students (US News, 2022). The demographic makeup of students was 46% Hispanic or Latino, 31% White, 6% American Indian or Alaska Native, 4% Asian, 4% Two or More Races, 3% Black or African American, and 0.2% Native Hawaiian or Other Pacific Islanders while 42% are male and 58% are female (Data US, 2022; US News, 2022). To our knowledge, there is not any publicly available data detailing student gender identity as all public datasets we could find solely detailed sex.

### Measures

Student demographics included race, gender, ethnicity, age, and degree. Transgender students and non-binary students were combined to form a larger transgender or gender diversity (TGD) category for meaningful comparison to cisgender students in analyses. We recognize, however, that transgender students and non-binary students are distinct groups with highly varied experiences and expression (Fiani & Han, 2020).

#### Clinical Measures

Clinical measures included the Patient Health Questionnaire 9 (PHQ-9) for depressive symptoms, the CAGE Substance Abuse Screening Tool for alcohol use and the PQ-B for symptoms of psychosis. The PHQ-9 consists of 9 items rated on 0- to 3-point Likert scales, which are summed to yield a composite score with higher scores indicating greater depressive symptom severity (Kroenke et al., 2001). In a sample of N = 277 depression patients, the PHQ-9 demonstrated adequate reliability and validity with *α* = 0.84, a sensitivity of 91%, and a specificity of 77% when using a cutoff of 5 (Sun et al., 2022). The CAGE consists of 4 yes or no items yielding a composite score ranging from 0 to 4 with higher scores indicating heightened alcohol use (Ewing, 1984). A recent Meta-Analysis found the CAGE has demonstrated adequate reliability and validity with a pooled *α* of 0.70, pooled sensitivity of 70%, and pooled specificity of 90% (Santos et al., 2020). All clinical measures were analyzed as continuous variables.

#### Screening Measure and Cutoff

The PQ-B consists of 21 yes or no items that describe positive symptoms of psychosis. Items that are endorsed are rated on 0- to 5-point Likert scales indicating the extent to which the symptom detailed is distressing to the respondent (Loewy et al., 2011). The sum of the endorsed items constitute the respondent’s total PQ-B score while the sum of the scores from the Likert scales completed constitutes the respondent’s distress score (Loewy et al., 2011). The PQ-B has demonstrated strong reliability for detecting symptoms of psychosis and related distress (Fonseca-Pedrero et al., 2016). In the study by Saavedra et al. (2023), two cutoffs were used, based on the phase of the study. During phase 1 (August 1, 2020–September 30, 2021), a PQ-B distress score cutoff of greater than or equal to 20 qualified students for referral to UNM’s CSC program. During phase 2 (October 1, 2021–May 13, 2022), a PQ-B distress cutoff of greater than or equal to 24 qualified students for referral.

Program specialists carried out the 15-min phone screening to assess the student’s psychosis symptoms (duration and severity), mental health history, medications, family history, and overall functioning as based on diagnostic criteria from the Structured Interview for Psychosis-Risk Syndromes (SIPS) (McGlashan et al., 2001). Next, clinicians administered the SIPS for students recommended for a CSC eligibility assessment after phone screening; however, some were directly enrolled in CSC services given their clinical history and current symptoms.

### Exclusion Criteria

For the current study, we were interested in students who would be eligible for CSC; therefore, this secondary analysis was restricted to students 30 years of age or younger to be in alignment with inclusion criteria for UNM’s CSC programs. For those who presented multiple times, we randomly selected one entry for inclusion in the final dataset. Sensitivity analyses (Fisher’s Exact tests for categorical variables and Wilcoxon rank-sum tests for continuous) comparing the demographic make-up and clinical characteristics of the whole sample before and after applying exclusion criteria revealed age as the only variable significantly impacted by the exclusion criteria (*p* < 0.001). The significant difference in age between the original study by Saavedra et al. (2023) and our sample was to be expected, given that we intentionally restricted the sample for this analysis to students 30 years of age or younger.

### Analyses

We first calculated counts to describe the demographic and clinical characteristics of students at each stage. We then assessed the relationship between demographic variables and engagement in the early detection cascade of care via Fisher’s Exact tests comparing students who reached the previous stage but did not advance to those who reached the previous stage and did advance. Three separate sets of Fisher’s Exact tests compared distributions of progression by racial/ethnic, gender, or degree groupings. Next, we assessed the significance of changes in clinical characteristics and age across stages via Wilcoxon rank-sum tests comparing scores of students screened via the PQ-B (stage 1) who reached each given stage versus those who reached the previous stage but did not reach the next given stage. Finally, we ran a logistic regression to assess which demographic and clinical variables predicted if students eligible for phone screening followed through or not (transition from stage 3 to 4). We included all variables significant at *p* < 0.10 as well as any confounding variables in our regression model (Bursac et al., 2008). All analyses were conducted using R (2023).

## Results

Table 1 describes the demographic and clinical makeup of students at each stage of the early detection cascade of care. Altogether, 280 students above 30 years of age were excluded from the analyses. During the study period, 62 students aged 30 years or younger presented to UNM Student Health and Counseling multiple times. Students screened via the PQ-B (stage 1) differed in demographic makeup from the general student population of UNM on all demographic variables. Of the students screened, 61% were women relative to 58% of the general UNM population, and students screened were 41% Hispanic or Latino relative to 46% of the general UNM population (Data US, 2022; US News, 2022). The following describes the number of students at each stage in the early detection cascade of care as a percentage of the stage directly before. Of the 1,588 students who completed the PQ-B at intake (stage 1), 486 (31%) met the PQ-B cutoff score (stage 2), 404 (83%) were referred for phone screening (stage 3), 198 (49%) completed phone screening (stage 4), 51 (26%) completed a CSC eligibility assessment (stage 5), and 21 (41%) were enrolled in treatment (stage 6).

**Table 1:** Demographic makeup of students at each stage of the early detection cascade

Stage	1 PQ-B Screening		2 PQ-B Cutoff Met		3 Phone screening referral		4 Phone screening		5 CSC eligibility screening		6 Enrollment in CSC	
	Count	Percent (%)	Count	Percent (%)	Count	Percent (%)	Count	Percent (%)	Count	Percent (%)	Count	Percent (%)
Total (percent of previous stage)	1588	100	486	31	404	83	198	49	51	26	21	41
*Race*												
White	1314	83	398	82	332	82	163	82	41	80	16	76
African-American and/or Black	68	4	20	4	18	4	9	5	3	6	0	0
Native-American	71	4	27	6	22	5	11	6	0	0	0	0
Asian and/or Pacific Islander	160	10	55	11	45	11	19	10	5	10	2	10
Multiracial	244	15	87	18	71	18	36	18	10	20	2	10
*Ethnicity*												
Hispanic and/or Latino	649	41	207	43	166	41	75	38	14	27	6	29
*Gender*												
Man	568	36	171	35	131	32	59	30	14	27	8	38
Woman	959	60	283	58	244	60	122	62	33	65	11	52
Cisgender	1503	95	442	91	370	90	174	88	45	88	18	86
Transgender and Gender Diverse	85	5	44	9	40	10	24	12	6	12	3	14
Non-Binary	59	4	32	7	29	7	17	9	4	8	2	10
Transgender	26	2	12	2	11	3	7	4	2	4	1	5
*Degree*												
Undergraduate	1120	71	399	82	339	84	162	82	46	90	18	86
Graduate	429	27	77	16	57	14	33	17	4	8	3	14

	Median	IQR	Median	IQR	Median	IQR	Median	IQR	Median	IQR	Median	IQR
Age	21.00	5.00	20.00	4.00	20.00	3.00	20.50	3.00	21.00	2.50	20.00	4.00
Clinical Characteristics												
CAGE	0.00	1.00	0.00	1.00	0.00	1.00	0.00	1.00	0.00	1.00	0.00	1.00
PHQ-9	11.00	9.00	15.00	8.00	15.00	8.00	15.00	8.00	18.00	8.00	17.00	12.00
PQ-B Total	4.00	6.00	9.00	3.50	9.00	3.00	9.00	3.75	10.00	4.00	9.00	5.00
PQ-B Distress	12.00	21.00	32.00	14.00	32.00	14.00	33.00	15.75	38.00	17.50	38.00	25.00

Categories for race, gender, and degree are overlapping given that many students identify with more than one of the listed demographic groups. This alongside some participants missing data for certain variables has led some variables to have counts for demographic groups that do not sum to the total number of participants at a given stage

Student gender identity and degree emerged as the only two demographic variables significantly associated with reaching later stages in the early detection cascade. Altogether, 52% of TGD students (n = 44 of 85) met the PQ-B cutoff necessary for further screening relative to 29% cisgender students (n = 442 of 1503, *p* < 0.001). Meanwhile 86% of women who met the PQ-B cutoff were referred for phone screening (n = 244 of 283) relative to 77% of men who met the PQ-B cutoff (n = 131 of 171, *p* = 0.016). Last, 36% of undergraduate students met the PQ-B cutoff necessary for further screening (vs. 18% of graduate students, *p* < 0.001), 85% of eligible undergraduates were referred for phone screening (vs. 74% of graduate students, *p* = 0.029), and 28% of eligible undergraduates completed a CSC eligibility assessment (vs. 12% of graduate students, *p* = 0.052). See Table 2 for the proportion of each demographic group screened via the PQ-B who reached each later stage and significance of all between groups differences.

**Table 2:** Transition rates between stages of demographic groups across the early detection cascade

Gender						
Stage	Men	Women	Significance (Fisher’s exact test)	Cisgender	Transgender and gender diverse	Significance (Fisher’s exact test)
1 PQ-B Screening	N = 568	N = 959	Men vs. Women	N = 1503	N = 85	TGD vs. Cisgender
2 PQ-B Cutoff Met	30% (N = 171)	30% (N = 283)	*p* = 0.817	29% (N = 442)	52% (N = 44)	***p* < 0.001**
3 Phone Screening Referral	77% (N = 131)	86% (N = 244)	***p* = 0.016**	84% (N = 370)	91% (N = 40)	*p* = .278
4 Phone Screening	45% (N = 59)	50% (N = 122)	*p* = 0.386	47% (N = 174)	60% (N = 24)	*p* = 0.135
5 CSC Eligibility Assessment	31% (N = 14)	27% (N = 33)	*p* = 0.719	26% (N = 45)	25% (N = 6)	*p* = 1.000
6 Enrollment in CSC	57% (N = 8)	33% (N = 11)	*p* = 0.195	40% (N = 18)	50% (N = 3)	*p* = 0.680

Degree			
Stage	Undergraduate	Graduate	Significance (Fisher’s exact test)
1 PQ-B Screening	N = 1120	N = 429	
2 PQ-B Cutoff Met	36% (N = 399)	18% (N = 77)	***p* < 0.001**
3 Phone Screening Referral	85% (N = 339)	74% (N = 57)	***p* = 0.029**
4 Phone Screening	48% (N = 162)	58% (N = 33)	*p* = 0.110
5 CSC Eligibility Assessment	28% (N = 46)	12% (N = 4)	***p* = 0.052**
6 Enrollment in CSC	39% (N = 18)	75% (N = 3)	*p* = 0.297

Race and ethnicity							
Stage	White	African-American and/or Black	Native-American	Asian and/or Pacific Islander	Hispanic and/or Latino	Multiracial	Significance (Fisher’s exact test)
1 PQ-B Screening	N = 1314	N = 68	N = 71	N = 160	N = 649	N = 244	
2 PQ-B Cutoff Met	31% (N = 398)	29% (N = 20)	39% (N = 27)	35% (N = 55)	32% (N = 277)	36% (N = 87)	*p* = 0.127
3 Phone Screening Referral	83% (N = 332)	90% (N = 18)	81% (N = 22)	81% (N = 45)	60% (N = 166)	82% (N = 71)	*p* = 0.361
4 Phone Screening	49% (N = 163)	50% (N = 9)	50% (N = 11)	42% (N = 19)	45% (N = 75)	51% (N = 36)	*p* = 0.351
5 CSC Eligibility Assessment	25% (N = 41)	33% (N = 3)	0% (N = 0)	26% (N = 5)	19% (N = 14)	28% (N = 10)	*p* = 0.483
6 Enrollment in CSC	39% (N = 16)	0% (N = 0)	0% (N = 0)	50% (N = 2)	43% (N = 6)	20% (N = 2)	*p* = 0.189

Percents listed were calculated by dividing the number of participants in each demographic group at a given stage by the number of participants in that demographic group in the previous stage. Significant between-groups difference are bolded. Significance of between-groups was calculated as based on non-overlapping groups as outlined in the analyses section.

P-values below 0.10 are bolded for easier viewing

Scores on the measures of alcohol use (CAGE) and depressive symptoms (PHQ-9) were higher, while age was lower, among students who met the PQ-B cutoff (all *p* < 0.001). PQ-B total and distress scores were higher (each *p* < 0.001) among students who were referred for phone screening, relative to those who met the PQ-B cutoff but were not referred. PQ-B total scores were higher (*p* < 0.001) among students referred for phone screening who completed it relative to those referred who did not. Finally, PQ-B distress scores (*p* = 0.010) and depressive symptomology (PHQ-9, *p* = 0.001) were higher among students who completed a CSC eligibility assessment, relative to students who did not complete a CSC eligibility assessment (See Table 3).

**Table 3:** Clinical makeup and age of students at each stage of the early detection cascade

Stage Transition	1–2 PQ-B Screening to Cutoff Met (n = 1588)			2–3 PQ-B Cutoff Met to Phone Screening Referral (n = 486)			3–4 Phone Screening Referral to Screening (n = 404)			4–5 Phone Screening to CSC Eligibility Assessment (n = 198)			5–6 CSC Eligibility Assessment to Enrollment (n = 51)		
Measure/Group	Reached Stage 2	Did Not Reach Stage 2	*p*	Reached Stage 3	Did Not Reach Stage 3	*p*	Reached Stage 4	Did Not Reach Stage 4	*p*	Reached Stage 5	Did Not Reach Stage 5	*p*	Reached Stage 6	Did Not Reach Stage 6	*p*
PQ-B Total Median (IQR)	9.0 (3.5)	2.0 (4.0)	** < 0.001**	9.0 (3.0)	8.0 (4.0)	** < 0.001**	9.0 (3.8)	9.0 (3.0)	** < 0.001**	10.0 (4.0)	9.0 (3.5)	0.342	9.0 (5.0)	10.0 (4.8)	0.030
PQ-B Distress Median (IQR)	32.0 (14.0)	6.0 (13.0)	** < 0.001**	32.0 (14.0)	27.0 (14.0)	** < 0.001**	33.0 (15.8)	32.0 (12.8)	0.390	38.0 (17.5)	32.0 (13.5)	**0.010**	38.0 (25.0)	36.5 (14.8)	0.653
CAGE Median (IQR)	0.0 (1.0)	0.0 (0.0)	** < 0.001**	0.0 (1.0)	0.0 (1.0)	0.550	0.0 (1.0)	0.0 (1.0)	0.070	0.0 (1.0)	0.0 (1.0)	**0.001**	0.0 (1.0)	0.0 (1.0)	0.931
PHQ-9 Median (IQR)	15.0 (8.0)	9.0 (8.0)	** < .001**	15.0 (8.0)	14.0 (6.0)	0.681	15.0 (8.0)	14.0 (8.0)	0.242	18.0 (8.0)	15.0 (6.5)	0.378	17.0 (12.0)	18.0 (6.8)	0.803
Age Median (IQR)	20.0 (4.0)	21.0 (5.0)	** < 0.001**	20.0 (3.0)	21.0 (4.0)	0.458	20.5 (3.0)	20.0 (3.0)	0.554	21.0 (2.5)	20.0 (4.0)	0.539	20.0 (4.0)	21.0 (2.0)	.830

Comparisons are between scores of those who progressed to the next stage vs. those who did not out of all participants in the previous stage

P-values below 0.10 are bolded for easier viewing

Our logistic regression model identified student gender identity, degree, alcohol use, and depressive symptoms as predictors for whether a student referred for phone screening followed through or not (See Table 4). Specifically, our model found evidence suggesting TGD students and weak evidence suggesting graduate students were more likely to follow through for phone screening than their counterparts (OR = 1.19, 95% CI = [1.00, 1.41], *p* < 0.05 and OR = 0.88, 95% CI = [0.77, 1.02], *p* < 0.10, respectively). Furthermore, our model found weak evidence suggesting that higher scores for depressive symptomology (PHQ-9) predicted follow-through on phone screening (OR = 1.01, 95% CI = [1.00, 1.02], *p* < 0.10) while lower scores for alcohol use (CAGE) predicted follow through (OR = 0.96, 95% CI = [0.92, 1.00], *p* < 0.10).

**Table 4:** Regression model of factors related to student follow through in secondary phone screening (stage 3–4)

Variable	Odds ratio (95% Confidence interval)
Intercept	1.63 (1.37, 1.94)****
^1^Gender Identity	1.19 (1.00, 1.41)**
^2^Education Level	0.88 (0.77, 1.02)*
CAGE (Alcohol Use)	0.96 (0.92, 1.00)*
PHQ-9 (Depressive Symptoms)	1.01 (1.00, 1.02)*
n	404

*p < 0.10, **p < 0.05, ***p < 0.01, ****p < 0.001

^1^Gender Identity: reference = Cisgender; ^2^Education Level: reference = Undergraduate

## Discussion

The present study sought to detail the demographic and clinical characteristics at each stage of the early detection cascade of care and to better understand what characteristics, if any, were associated with progression between stages. While the overall sample of students who presented to SHAC did not differ from the general student population at UNM, we did find that undergraduate degree, TGD gender identity, heightened alcohol use, and heightened depressive symptoms were significantly associated with progression through at least one transition in the early detection cascade. Further examination via logistic regression (a separate analysis) of the transition with the highest disengagement (stage 3–4) through logistic regression, revealed that TGD gender identity significantly predicted if students referred for phone screening followed through or not.

The finding that TGD students were significantly more likely than cisgender students to progress through the early detection cascade at specific stages, including the student dependent stages of following up for a phone screen, was unexpected and warrants further discussion. Little is known about the pathways to care for and treatment of psychosis in gender diverse populations despite TGD populations being at greater risk for psychiatric disorders and suicide (Barr et al., 2021). Although TGD students transitioned to almost all subsequent stages at higher rates relative to cisgender students, this difference was only significant in the case of PQ-B screening outcomes (transition from stage 1 to 2) in which 52% (44 of 85) of TGD students met the cutoff for further screening relative to 29% of cisgender students (442 of 1503). TGD students may have scored higher on the PQ-B due to wording of items, which may have misattributed gender dysphoria for symptoms of psychosis. For example, Item 16 of the PQ-B asked “Do you feel that parts of your body have changed in some way, or that parts of your body are working differently?” In addition, it is possible that TGD students were more likely than their cisgender counterparts to progress through the early detection cascade due to clinical over-pathologizing or bias by college counselors who were responsible for making referrals for a secondary phone screen based on the results of the PQ-B. However, given the relatively small number of TGD students in stages following PQ-B screening, we cannot be confident heightened transition rates among TGD students at later stages are not due to chance.

Interestingly, clinicians referred a significantly higher proportion of women who met the PQ-B cutoff for phone screening at 86% (244 of 283) relative to 77% (131 of 171) of men who met the PQ-B cutoff for phone screening (transition from stage 2–3). While women are twice as likely as men to seek out mental health treatment (Fridgen et al., 2013), research has repeatedly found men to have a higher incidence of psychotic disorders than women (Ochoa et al., 2012). Noteworthy, the higher incidence of psychosis among men compared to women has been attributed to heightened cannabis use among men (Riecher-Rössler et al., 2018)). Counselors may not have referred some of the men who met the PQ-B cutoff for further screening if they thought substance use focused treatment would have been more beneficial than a referral to CSC.

Overall, undergraduates were more likely to meet the PQ-B cutoff, be referred for phone screening, and complete a CSC eligibility assessment (reaching stages 2, 3, and 5 respectively) when compared to graduate students. Graduate students may have engaged in later stages of the early detection cascade at slower rates given that individuals above the age of 25 who did not meet diagnostic criteria for FEP but did meet diagnostic criteria for clinical high risk were ineligible for services due to UNM’s CSC for CHR only treating individuals 25 years of age or younger. However, findings cannot be attributed solely to age given that only meeting the PQ-B cutoff (reaching stage 2) was significantly associated with age. There is a possibility that undergraduate students’ increased engagement in the early detection cascade can be accounted for by greater distress and help-seeking behaviors associated with the transition to independent college life (Pedrelli et al., 2015).

Our finding that students with higher scores for depressive symptomology (PHQ-9) and lower scores for alcohol use (CAGE) were more likely to follow-through with referral for a secondary assessment is consistent with common patterns in mental health service engagement. Specifically, research has demonstrated that college students with mental health problems, such as depression, tend to seek treatment more than those students with alcohol use related problems (Blanco et al., 2008; Kenney et al., 2018).

### Limitations

Our study examined whether race, gender, ethnicity, age, degree, alcohol use, depressive symptomology and scores on the PQ-B were related to progression in the early detection cascade of care. However, there are additional student and program-level factors that likely influence engagement in the cascade. For example, student level factors could also include financial concerns, insurance type, transportation, and lack of information about services as barriers to engaging in psychiatric services (Wang et al., 2020). Other program level factors include, for example, bias associated with counselor referral patterns and determinations regarding the need for further assessment. The current study did not include collection of data for these potential factors.

Another limitation is that we categorized transgender and non-binary students into a singular group of TGD students given that TGD students made up a small portion (85 of 1588; 5.4%) of our total sample. Existing general mental health research focuses on TGD populations as a whole or solely transgender populations (Connolly et al., 2016). Further research with larger samples is needed to determine if outcomes differ for non-binary vs. transgender young adults at-risk for and experiencing psychosis.

Finally, the study population (i.e., college students) may not be fully representative of CHR or FEP young adult populations. College students may have distinct and non-generalizable reasons for engagement/disengagement in an early psychosis detection program such as increased access to resources compared to the general population. In addition, the original study conducted by Saavedra et al. (2023) occurred during the COVID-19 pandemic and research has shown that college students were negatively impacted during this time, including increased levels of fear, stress, mental health problems and decreased happiness and access to care (Wang et al., 2020). As a result, the (1) nature of and severity of early psychosis, alcohol use and depressive symptoms experienced among our population, (2) barriers faced in being connected with screening or services, and (3) types of students who engaged in screening or services might not be representative of college students post-pandemic.

## Conclusion

This exploratory study examined if certain demographic and clinical characteristics were associated with students disengaging from the early detection cascade of care. Results from this study have implications for targeted strategies that could be developed to reduce disengagement and potentially shorten DUP. With this in mind, the finding that graduate students were more likely to disengage a majority of transitions in the early detection cascade suggests the importance of targeted efforts to decrease this subgroup of students’ likelihood of falling “through the cracks”. The finding that TGD students were more likely to meet the PQ-B cutoff warrants evaluation of how scores on specific items and factors of the PQ-B may differ between cisgender and TGD young adults. Currently, little to no research exists detailing any relationships between gender and psychosis screening besides comparing scores of men and women (Bridgwater et al., 2023). Future research also needs to explore how other student- and program-level factors, not assessed here, may explain why some students who meet screening cutoffs are not referred or are referred and disengage.
